# Self-assessment of olfactory function using the “Sniffin’ Sticks”

**DOI:** 10.1007/s00405-023-07872-7

**Published:** 2023-02-17

**Authors:** Yiling Mai, Marie Klockow, Antje Haehner, Thomas Hummel

**Affiliations:** Department of Otorhinolaryngology, Smell and Taste Clinic, Technical University of Dresden, Fetscherstrasse 74, 01307 Dresden, Germany

**Keywords:** Self-assessment, Self-test, Sniffin’ Sticks, Olfactory dysfunction, Test–retest reliability

## Abstract

**Background:**

A precise and reliable test of the olfactory function is indispensable for the diagnosis of the olfactory disorder (OD). Despite of this, in a clinical context, often there is no place in daily routine for time-consuming procedures. This study aimed to examine if the assessment of olfactory function using the “Sniffin’ Sticks” is suitable for self-assessment.

**Methods:**

Participants comprised 84 healthy control subjects (HC) and 37 OD patients. The “Sniffin’ Sticks” test battery consisting of odor threshold (T), discrimination (D) and identification (I) tests was used for self- and assisted assessments. To save time, we applied the 8-item wide step version of the T test and the 8-item D test, whereas the I task remained the same as the original version. The whole test included two sessions, with each session comprising a self-assessment part performed by the participants themselves, and an assisted-assessment part performed by the examiner.

**Results:**

Sniffin’ Sticks self-assessment was efficient in distinguishing between self-reported HC subjects and OD patients (*p*’s < 0.01), and the scores did not differ significantly from the assisted-assessment (*p*’s > 0.05). In the self-administered I and TDI tests, there was a moderate to excellent test–retest reliability (ICC = 0.51–0.93, *p*’s < 0.01), and a strong to excellent correlation with the assisted assessment (*r* = 0.71–0.92, *p*’s < 0.01). However, the self-administered T and D tests only exhibited low to moderate test–retest reliability (ICC = 0.30–0.72, *p*’s < 0.05) and correlations with the assisted test (*r* = 0.31–0.62, *p*’s < 0.05).

**Conclusions:**

The Identification self-test is appropriate to be solely applied, and is therefore an easy-to-use alternative for olfactory screening in a larger segment of patients. The whole “Sniffin’ Sticks” self-test also shows good measurement properties and is therefore a suitable backup in clinical practice, but improvement is needed due to the simplified D and T self-test.

## Background

Olfactory dysfunction (OD) is a common disorder, with a prevalence of approximately 22% among the general population [1]. It is not only an early sign of neurodegenerative diseases, including Alzheimer’s disease and Parkinson’s disease, but also closely related to many serious medical outcomes such as obesity [2], malnutrition [3], schizophrenia [4], or depression [5]. Extensive evidence also suggests that OD has a negative impact on quality of life [6] and psychological well-being [7]. Hence, it is important to offer therapeutic options and counseling to OD patients, starting with a precise and reliable assessment of the disorder.

Clinically, olfactory tests usually utilize psychophysical methods to assess subjects’ ability to detect, discriminate, or identify odors. Olfactory threshold tests measure the lowest concentration of an odor that a subject can perceive, while discrimination tests evaluate the subjects’ ability to distinguish different odors. As for odor identification tests, it assesses one’s ability to recognize an odor using a list of descriptors [8]. One of the most frequently used tests in the world is the “Sniffin’ Sticks” test [9]. It comprises Threshold, Discrimination and Identification tests, and allows to sum all the three dimensions to one score (TDI) that reflects the overall olfactory function. In Threshold test, participants always receive a sequence of three pens with two of those pens containing odorless solvent, and the third pen containing the odorant. The participants have to identify the odorous pen even if they are not certain about their sensations. A staircase paradigm is performed where two subsequent correct identifications of the odorous pen or one incorrect answer trigger a turning point, and result in a decrease or increase, respectively, of the odor concentration applied in the next triplet [10]. There are 16 concentrations in total, and the test starts from the lowest concentration. The threshold score is the mean of the last four turning points in the staircase [11]. In Discrimination test, there are 16 triplets of odorous pens. Within each triplet of pens, two of them contain the same odor and the third pen contains a different odor. Participants are required to identify the odd one [11]. In the identification test, there are 16 odorous pen that filled with different odorants. Participants are presented one odorous pen each time, and asked to select one of the four items from the flash cards that best describe the odor [11]. The Sniffin’ Sticks test is widely-used and well-validated. However, the use of the “Sniffin’ Sticks” mainly relies on the assistance of an examiner in both clinical and research settings [12]. This limits the utilization of the test when personnel resources are limited in clinical routine. Thus, developing and validating a self-administered test is meaningful in the routine situation of a busy ENT practice [13].

Previous studies developed tests suitable for self-assessment of olfactory function. However, some of them aimed specifically for the screening of subjects using household materials that is limited in terms of control of odor concentrations and the quality of the odors used [13]. Other studies, although developed on the basis of well-validated tools, did not address the complete range of olfactory functions (T, D and I), that could provide more precise information for the diagnosis of olfactory disorder compared to a single aspect of the olfactory function [14]. Two studies developed the “odor-curves-on-paper” method using the odorous pen to draw a line on a paper and then smell the odor from the paper as a self-test procedure to assess the olfactory function [12, 15]. However, it is likely that the odor concentrations presented from the paper are significantly lower than odor concentrations presented from the tip of the pen tip, rendering the test significantly more difficult in patients with olfactory dysfunction. In addition, the test–retest reliability is not known [16]. Last but not least, most of the previous studies did not investigate the participants’ attitude towards self-assessment, which is often a critical issue whether a tool is widely accepted or not.

Based on the limitations in these previous studies, the present investigation aimed to highlight several points important for the development of a self-administered olfactory test. The test to be developed should (1) have good test–retest reliability and inter-test consistency, (2) assess complete olfactory function, i.e., T, D, and I, (3) be time-effective and easily-applicable, (4) be widely available, and (5) be accepted by the participants. Because the Sniffin’ Sticks test battery is well validated for its assisted administration and is widely-used in ENT clinics, it is promising to develop a self-administered procedure for the Sniffin’ Stick test. Our present study therefore aimed at examining if the complete assessment of olfactory function using the “Sniffin’ Sticks” is reliable and suitable for self-assessment.

Specifically, our study had 4 aims: (1) develop a self-administered procedure of Sniffin’ Sticks test by giving appropriate instructions to the participants; (2) assess the test–retest reliability by adding a follow-up self-assessment in each participant; (3) determine the inter-test consistency by adding an assisted-assessment for inter-test comparison; (4) explore participants’ attitude toward self-assessment. Notably, due to the staircase procedure in Threshold test, it is impossible for participants to conduct the test themselves. Hence, an examiner or adaptive software is needed to provide “real-time” instructions (i.e., guide the participants which concentration [labeled as number] of the pen triplet they should take). Therefore, within the context of the present study “self-administered” refers to “assisted self-assessment”. In addition, due to our purpose of performing both self- and assisted-assessment for inter-test comparison, the required test time of completing both tests would double. To balance the effectiveness of the test and attention and task burden of the participants, it appeared to be important to shorten parts of the Sniffin’ Sticks test procedure: (1) for the Threshold test, the most time-consuming and complex subtest, an 8-item wide-step version would be suitable, because it has been shown to save testing time and to yield reproducible results [17]. (2) For the Discrimination test, an 8-item version was used, where items were randomly selected from the original 16-items version (participants need to smell 48 odors for a single D test); (3) for the Identification test, because the test itself is relatively entertaining and it typically does not take long, it appeared not to be necessary to change the Identification test.

## Methods

### Participants

In the current study, 121 participants were included as a sample of convenience. There were 37 patients with subjective complaints of olfactory disorders (OD) recruited from the Smell and Taste Clinic, Department of Otorhinolaryngology, Technische Universität Dresden, Germany. All patients were diagnosed according to the current diagnostic ENT criteria for smell disorders, including anterior rhinoscopy, nasal endoscopy, and olfactory testing (standard Sniffin’ Sticks test) which ensures correct diagnosis assignment [18]. There were also 84 adult participants with a self-reported normal sense of smell recruiting as healthy control (HC) group. The design of this study was approved by the Ethics committee at the Medical Faculty of the TU Dresden (application number EK 156052012). All participants provided written informed consent.

### Procedure

The test included two sessions using the same procedure. Each session included an active, self-administered Sniffin’ Stick test performed by the participants themselves, as well as the passive, assisted-administered Sniffin’ Sticks test performed by the examiner. In session 1, the examiner started administering the test for the participants, and then the participants applied the test themselves according to the instructions. The instructions could be provided by the examiner or a computerized program. In current study, the focus was on the question whether the self-assessment could be reliably and validly applied, and the form as to how give instructions was not the main issue of the present study. Hence, in current study guidance was provided by the examiner. In session 2, the subjects first administered the test themselves, then the test was performed by the examiner. To be specific, the sequence of the self- and assisted-administered tests were as follows. Session one: I assisted, I self; D assisted, D self; T assisted, T self. Session two: I self, I assisted; D self, D assisted; T self, T assisted. The test started with the easiest part (Identification) to show the participants how to handle the pens properly, so that they could get a feel for the procedure before moving on to the more difficult part of Threshold testing. Because participants performed the self-test part with eyes open, cap colors in the Threshold and Discrimination test were randomized and the answers were coded before testing. By this way, participants were prevented from guessing the answer.

In addition, participants were asked not to touch their noses with the tip of the pens. However, it was assumed that this instruction might not prevent participants from touching their nose. To control for possible microbial contamination, microbiological testing of nine randomly selected pens from the modified test kit was performed twice during the course of the study. The first screening at the beginning of testing was repeated after one year of regular use for self- and assisted-assessment, both showing no indication of pathological bacterial or fungal contamination of the pens. Hence, we assessed the infectiological risk to be reasonably low.

Participants had to restrain from eating or drinking anything but water for at least 30 min prior to testing [12]. The exact time required for the test was not recorded, but it took approximately 45–60 min for each session, including assisted and self-assessments. It took about half of the time to complete each part, but the assisted-test part was slightly shorter than the self-test part.

### Measurements

#### Sniffin’s Stick

The “Sniffin’ Sticks” (Burghart, Holm, Germany) comprises odor threshold (T), discrimination (D) and identification (I) was used for current study.

For the T test, a wide step method was used, with only 8 dilutions but covering the same range of concentrations of the regular 16 dilutions version [17]. Eight concentrations were created by first building the original 16 concentrations with a dilution ratio of 1:2 (narrow step method starting from a 4% solution), and then every second step of the narrow step method was left out, so that 8 different dilutions remained in the wide step method. The threshold score was the mean of the last two turning points in the staircase, ranging from 1 to 8 points [17]. Using the staircase paradigm (see above) in the assisted test part, the examiner conducted the test by presenting the participants a sequence of three pens, and asking them to identify the odorous pen from two odorless pens. In the self-test part, the participants were guided by the examiner which triplet of pens they should take. They then smelled the three pens one by one and tell the examiner, which of them contained the rose-odor. Depending on the answer, the examiner would tell the participants which triplets to take next and, by that, guide them through the test.

For the D test, a simplified version with half of the items (8 items) of the original test was used to save time. Subjects had to distinguish the target odor from two identical odors. Targeted odorous pens were randomly color-coded. The D score was the sum of all correctly identified odors, ranging from 0 to 8 points [11]. The part of assisted-testing was conducted by the examiner. While the self-administered part was performed by the participants themselves by taking the triplets with the same number (from 1 to 8), distinguishing one target odor from two identical odors and report the answer to the examiner.

For the I task, participants were presented one odorous pen each time by the examiner (assisted-test) or themselves (self-test). They then selected one of the four descriptors from the flash cards that best described the odor and reported the answer to the examiner for documentation. The I test score was the sum of all correctly identified odors, ranging from 0 to 16 points [11]. The final “TDI score” was the sum of scores for the I, D and T subtests [11], ranging from 1 to 32 points in the present study.

#### Convenience and pleasantness of the tests

After each subtest, participants were asked to compare self- and assisted-test as to pleasantness and convenience from a single question “Which test is more pleasant for you?”, and choose one alternative from “self”, “assisted” and “equal”.

### Statistical analyses

Data were analyzed by means of SPSS 27 software (IBM Corp., Armonk, NY, USA). We first conducted descriptive analyses to describe the demographic information of the full sample as well as the clinical characteristics of the OD patients.

Next, to check self- and assisted-test consistency, Person’s correlation between self- and assisted-test were adopted and interpreted as follows: *r* ≥ 0.9 indicates perfect correlation, 0.7 ≤ *r* < 0.9 indicates strong correlation, and 0.4 ≤ *r* < 0.7 indicates moderate correlation [19]. And dependent t tests were also conducted to check self- and assisted-test consistency in the total sample and two participants groups. In addition, the Bland–Altman plot with the mean and 95% limits of agreement (LOA) were reported to complement test interpretation [20]. Furthermore, as a supplementary analysis, to check whether the self-administered test was consistent with assisted testing, we calculated the error rate of the test. Error was defined as the score of the inter-test difference which exceeded the Minimum Clinically Important Difference (MCID) for the TDI, T, D, and I test, respectively. MCID represents the smallest change in a treatment outcome that an individual patient would identify as a noticeable and significant change [21]. If the self- test and assisted-test are consistent, the inter-test difference should be within the range of MCID. If the difference is greater than MCID the two methods are not statistically identical. In previous studies, MCID of the Sniffin’ Stick test was the repeated-test difference that 60% [22] of the participants rated as an improvement after treatment. We thus predefined a maximal acceptable error rate as 40% [22]. That means, if no more than 40% of the participants showed a change between self- and assisted-test, greater than the MCID, we could assume that the test tool exhibits an acceptable degree of reliability. According to previous studies, MCID for the TDI score is 5.5, it is 3 for the I test, 3 for D, and 2.5 for T [22]. To make the present test comparable with the corresponding MCID, transformation was needed. The self-test TDI scores that ranged from 1 to 32 were transformed to the range of the standard TDI score, from 1 to 48 (TDI_transformed_ = T_self_ × 2 + D_self_ × 2 + I_self_). The self-T, and self-D scores that ranged from 1 to 8 were transformed to the range of 1–16 (T_transformed_ = T_self_ × 2, D_transformed_ = D_self_ × 2).

Third, to assess test–retest reliability, intraclass correlation coefficient (ICC) were calculated [23, 24]. Because the interval between test and retest varied, we further calculated test–retest reliability for tests with “short” intervals (≤ 2 weeks) and tests performed at “longer” intervals (> 2 weeks), respectively. Generally, ICC ≥ 0.9 indicates excellent reliability, 0.75 ≤ ICC < 0.9 indicates good reliability, and 0.5 ≤ ICC < 0.75 indicates moderate reliability [25]. Similarly, the Bland–Altman plot with the mean and 95% limits of agreement (LOA) were reported to complement test interpretation [20]. And the error rate was calculated in the same way of what mentioned above to check if the self-test method is reliable and stable enough.

In addition, to examine if Sniffin’ Sticks self-assessment could distinguish between OD and HC, three-way repeated measures ANOVA (rmANOVA) analyses with Bonferroni post-hoc tests were conducted, with group (HC and OD) setting as between-subject factor, session (1 and 2) and test (T, D, I) setting as within-subject factors. Two-way rmANOVA with Bonferroni post-hoc tests were also conducted for self-assessed TDI total score, with group setting as between-subject factor and session as within-subject factor.

Last but not least, the percentage of subjects rating self-assessment and assisted-assessment as more convenient/pleasant were computed and described. The alpha level was set at 0.05.

## Results

### Descriptive analyses

Overall, we included 121 participants (75 women) aged 19–94 years with an average age of 41.3 ± 19.7 years old. The test–retest time interval ranged from 1 to 475 days with the medium ± interquartile range (IQR) of the retest interval of 18 ± 95 days. There were 54 and 67 participants, respectively, who had the retest interval ≤ 14 days and > 14 days, respectively. Among all the participants, 37 (16 women) were patients with olfactory disorder (OD) aged between 19 and 94 years with an average age of 46 ± 18 years, and 84 (59 women) were healthy controls (HC) aged between 19 and 79 years with an average age of 39 ± 20 years old. No significant difference in terms of age between HC and OD group (*t* = 1.67, *p* = 0.10) was found. However, there was a significant difference in terms of sex distribution between OD and HC group, with higher proportion of female participants (70%) in HC group compared to the proportion of female participants (43%) in OD group (*χ*^2^ = 7.94, *p* < 0.01). Clinical features of OD patients are shown in Table 1.

**Table 1:** Descriptive results of all participants and clinical features of patients with olfactory disorder

	OD (*N* = 37)	HC (*n* = 84)	Total sample (*n* = 121)	*t*/*χ*^2^	*p*
Age	45.7 ± 18.4	39.3 ± 20.0	41.3 ± 19.7	1.67	0.10
Gender					
Women	16 (43%)	59 (70%)	75 (62%)	7.94	< 0.01
Men	21 (57%)	25 (30%)	46 (38%)		
Test–retest time interval (days)	11 ± 55	42 ± 210	18 ± 95		
≤ 14 days	8 (22%)	46 (55%)	54 (45%)		
> 14 days	29 (78%)	38 (45%)	67 (55%)		
Causes					
Viral infections of the upper respiratory tract	14 (38%)				
Sinonasal	10 (27%)				
Idiopathic	9 (24%)				
Head trauma	2 (5%)				
Post-operative (neurosurgery and maxillofacial surgery)	1 (3%)				
Congenital	1 (3%)				
Disease duration (months)	31.5 ± 41.5				
Olfactory function	21.8 ± 7.6				
Anosmia	12 (32%)				
Hyposmia	18 (49%)				
Normosmia	7 (19%)				

Olfactory function tested using the standard version of Sniffin’ Stick test battery

*OD* olfactory disorder, *HC* healthy control

*p < 0.05, **p < 0.01

### Inter-test consistency

**Fig. 1:**
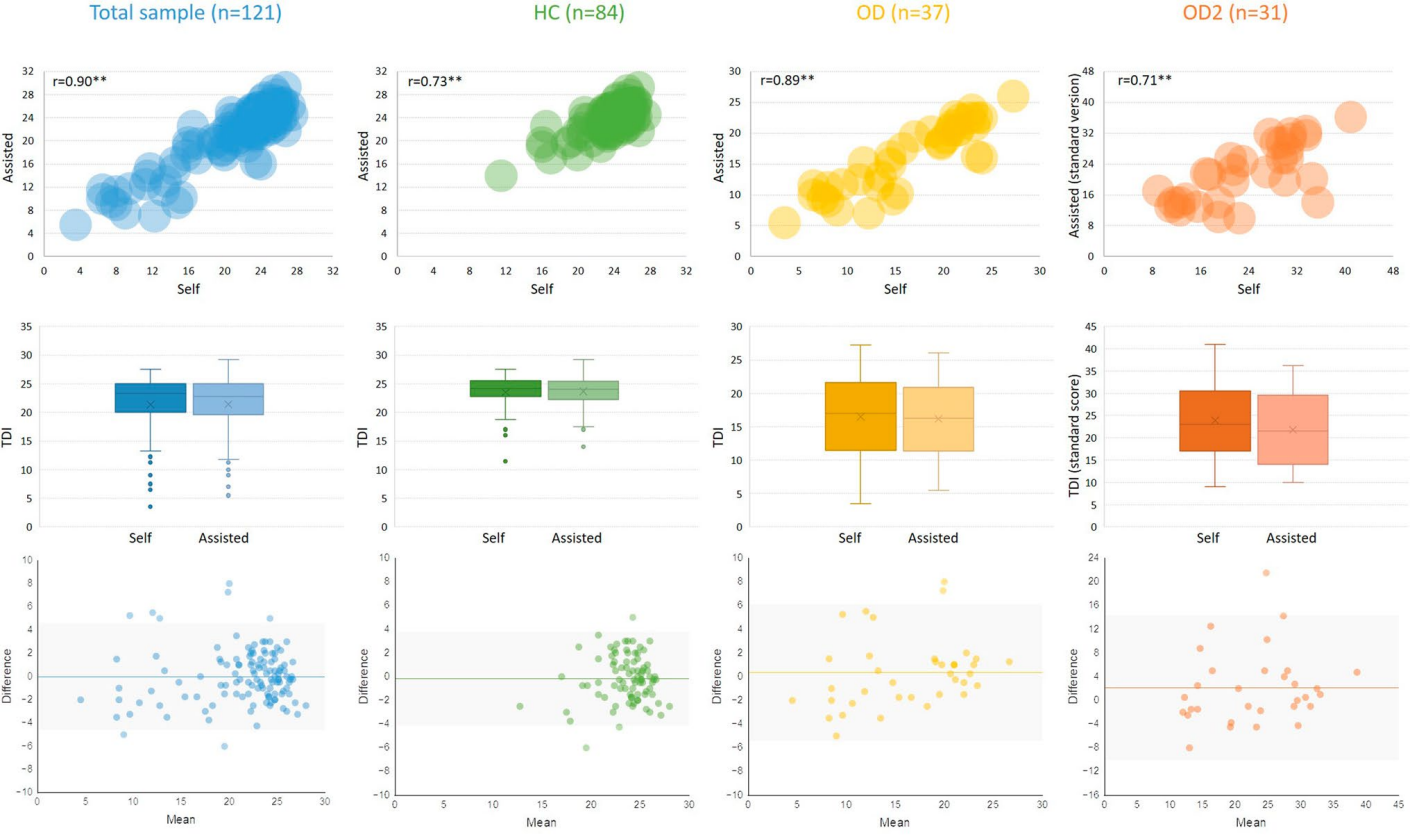
Bubble scatter plots, box plots and Bland–Altman plots of TDI scores between the assisted and self-test of the “Sniffin’ Sticks” First row = bubble scatter plots; second row = box plots; third row = Bland–Altman plots. OD = olfactory disorder group; HC = healthy control group. OD2 = OD group that had been tested with standard Sniffin’ Sticks. Before compared the self-test TDI score to the standard assisted TDI score in OD2, the self-test TDI scores that ranged from 1 to 32 were transformed to the range of the standard TDI score, from 1 to 48 (TDI_transformed_ = T_self_ × 2 + D_self_ × 2 + I_self_). The boxes indicate the interquartile range (IQR), with a horizontal line representing the median value and a cross representing the mean value. Values within upper and lower whiskers are highest and lowest data points in the data set excluding any outliers. Outliers (1.5 IQR above the third quartile) are shown in dots. Difference = Differences of scores between the first and second session, Mean = Mean scores from the first and second session. 95% limits of agreement (LOA) are indicated within the grey area, mean difference is indicated by the horizontal line. The number of data points superimposed on each other is indicated by the shade of the color—the more data points on top of each other the darker the color

As shown in Fig. 1, there were statistically consistent TDI scores between assisted tests and self-assessments in the total sample (21.37 ± 5.29 vs*.* 21.38 ± 5.12, *t* = 0.05, *p* = 0.96), patient group (16.48 ± 6.31 vs*.* 16.16 ± 5.56, *t* = 0.67, *p* = 0.51), and control group (23.52 ± 2.79 vs*.* 23.68 ± 2.64, *t* = 0.72, *p* = 0.48). In addition, for OD group who have been tested with the standard version of the Sniffin’ Sticks before being included in the cohort, we also compared their self-test TDI score to the standard assisted TDI score. Before comparison, the self-test TDI scores that ranged from 1 to 32 were transformed to the range of the standard TDI score, from 1 to 48 (TDI_transformed_ = T_self_ × 2 + D_self_ × 2 + I_self_). As a result, there was a statistically identical TDI score between self-test and the standard assisted test (23.89 ± 8.55 vs. 21.79 ± 7.64, *t* = 1.88, *p* = 0.07).

Regarding inter-test correlation, there were significant and positively strong correlations of TDI and I scores between self- and assisted assessments in the total sample, HC, and OD groups (r ranged from 0.71 to 0.92, *p*’s < 0.01). As for T and D tests, significantly moderate correlations between self- and assisted-assessments were found in the total sample and OD group (r ranged from 0.51 to 0.62, *p*’s < 0.01). However, correlations of T and D scores between self- and assisted assessments were statically significant but even lower than the acceptable level of 0.4 in HC group and OD group who had been tested with the standard version of the Sniffin’ Sticks (r ranged from 0.31 to 0.41, *p*’s < 0.05). See Table 2 and Fig. 1.

**Table 2:** Inter-test correlations between self- and assisted assessment

	Total sample (*N* = 121)	HC (*N* = 84)	OD (*N* = 37)	OD^a^ (*N* = 31)
TDI	0.90**	0.73**	0.89**	0.71**
I	0.92**	0.82**	0.89**	0.82**
D	0.62**	0.36**	0.61**	0.37*
T	0.51**	0.31**	0.51**	0.41*

*r* ≥ 0.9 indicates perfect correlation, 0.7 ≤ *r* < 0.9 indicates strong correlation, and 0.4 ≤ *r* < 0.7 indicates moderate correlation. I = Sniffin’ Stick Identification test. D = a simplified version with half of the items (8 items) of the standard Sniffin’ Stick Discrimination test. T = a wide step method with 8 dilutions but covering the same range of concentration of the standard Sniffin’ Stick Threshold test. a = correlations of OD group between simplified self-assessment and standard assisted assessment of Sniffin' Stick

*p < 0.05, **p < 0.01

Bland–Altman plots of TDI scores between self- and assisted-assessment were shown to complement test interpretation [20]. For the total sample, the mean difference was − 0.01, 95% LOA ranged from − 4.58 to 4.56. For the HC group, the mean difference was − 0.16, 95% LOA ranged from − 4.11 to 3.79. For the OD group, the mean difference was 0.32, 95% LOA ranged from − 5.43 to 6.08. For the OD group who had standard assisted TDI score, the mean difference between self-test and standard assisted-TDI score (transformed before comparison) was − 2.10, 95% LOA ranged from − 10.10 to 14.29 (See Fig. 1).

In addition, the rate of inter-test differences that exceed the corresponding MCID were reported. The inter-test error rates of TDI and I score in all groups were less than the predefined maximal error rate (40%): 16–19% of the TDI test, and 1–19% of the I subtest. When it comes to D and T subtest, inter-test error rates were within the range of 29–38% in the total sample and OD group. However, the error rates increased or even exceed the maximal acceptable error rate of 40% in the HC group and in the OD group who had a standard assisted Sniffin’ Sticks testing (25–61%). See Table 3.

**Table 3:** Percentage of participants that inter-test difference (error) reaches clinical significance (MCID)

	Error rate (|MD_self-assisted_|> MCID)			
	Total sample (*N* = 121)	HC (*N* = 84)	OD (*N* = 37)	OD^a^ (*N* = 31)
TDI	20 (17%)	13 (16%)	7 (19%)	6 (19%)
I	8 (7%)	1 (1%)	7 (19%)	5 (16%)
D	46 (38%)	21 (25%)	14 (38%)	13 (42%)
T	35 (29%)	34 (41%)	12 (32%)	19 (61%)

Error rate = Percentage of participants who had a between-test difference greater than the minimum clinically important difference (MCID) of the corresponding Sniffin’ Stick test. MCID for TDI was 5.5, I was 3, for D was 3 and for T was 2.5 [22]. Maximally clinical acceptable error rate was defined as 40% [22]. For calculating error rate, the self-test TDI scores that ranged from 1 to 32 were transformed to the range of the standard TDI score, from 1 to 48 (TDI_transformed_ = T_self_ × 2 + D_self_ × 2 + I_self_). The self-T, and self-D scores that ranged from 1 to 8 were transformed to the range of 1 to 16 (T_transformed_ = T_self_ × 2, D_transformed_ = D_self_ × 2)

### Test–retest reliability

**Fig. 2:**
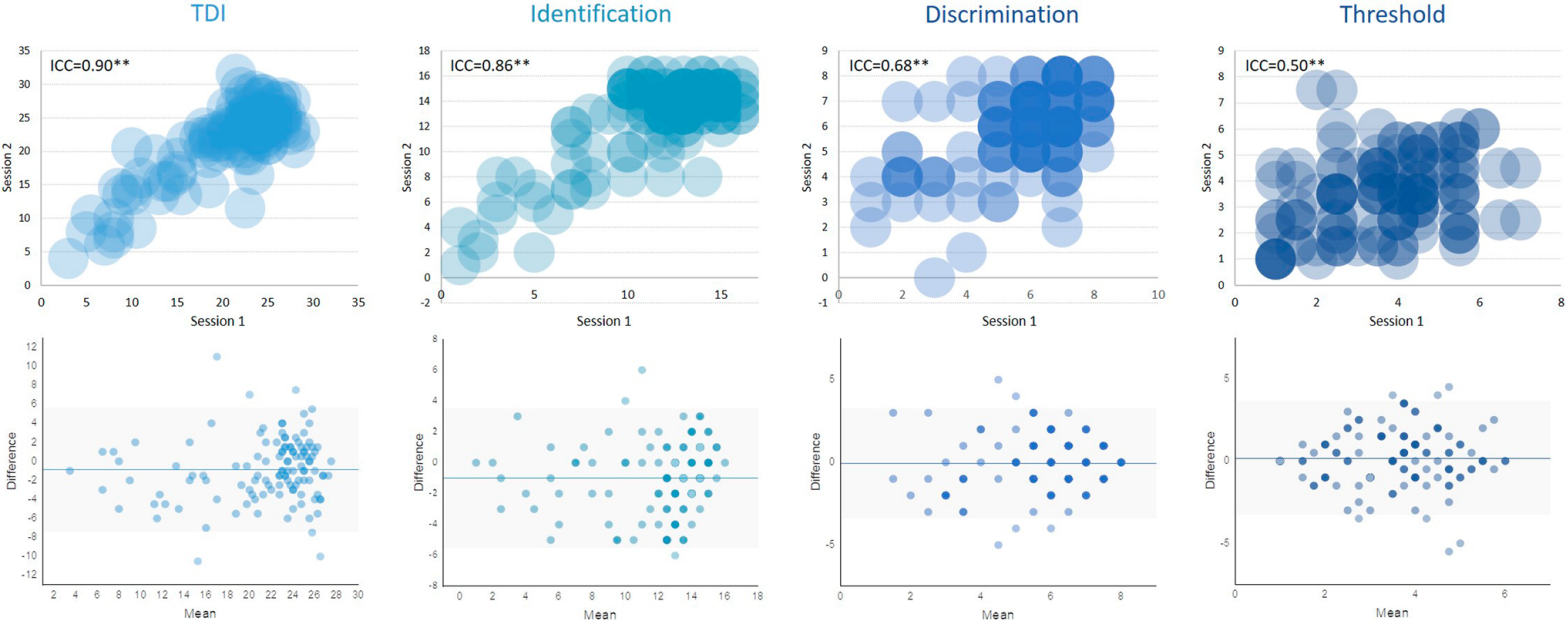
Bubble scatter plots and Bland–Altman plots of self-test of “Sniffin’ Sticks”. Bubble scatter plots (first row) and Bland–Altman plots (second row) of TDI-scores and scores for odor identification (I), odor discrimination (D) and odor threshold (T) between the first and second visits in the total sample. ICC = intraclass correlation coefficients, an indicator of test–retest reliability. Asterisks indicate significant results (**p < 0.01). The number of data points superimposed on each other is indicated by the shade of the color—the more data points on top of each other the darker the color. Difference = Differences of scores between the first and second session, Mean = Mean scores from the first and second session. 95% limits of agreement (LOA) are indicated within the grey area, mean difference is indicated by the horizontal blue line

As shown in Table 4 and Fig. 2, test–retest reliability of Sniffin’ Sticks self-assessment in total sample were ICC = 0.90, *p* < 0.01 (TDI); ICC = 0.86, *p* < 0.01 (I); ICC = 0.68, *p* < 0.01 (D); ICC = 0.50, *p* < 0.01 (T). For OD group, ICC = 0.93, *p* < 0.01 (TDI); ICC = 0.88, *p* < 0.01 (I); ICC = 0.72, *p* < 0.01 (D); ICC = 0.61, *p* < 0.01 (T). For HC group, ICC were much smaller: ICC = 0.61, *p* < 0.01 (TDI); ICC = 0.51, *p* < 0.01 (I); ICC = 0.40, *p* = 0.01 (D); ICC = 0.30, *p* = 0.50 (T). Because of the variation in the test–retest time interval, we further calculated test–retest reliability for “short” intervals (≤ 2 weeks) and “long” intervals (> 2 weeks), respectively (Table 4).

**Table 4:** Test–retest reliability of the self- and assisted test of “Sniffin’ Sticks”

	ICC		
	All subject (*n* = 121)	= < 2 weeks (*n* = 54)	> 2 weeks (*n* = 67)
Total sample			
Self			
TDI	0.90**	0.88**	0.90**
I	0.86**	0.86**	0.85**
D	0.68**	0.69**	0.68**
T	0.50**	0.40*	0.56**
Assisted			
TDI	0.90**	0.89**	0.90**
I	0.89**	0.89**	0.88**
D	0.64**	0.55**	0.69**
T	0.73**	0.66**	0.75**

	ICC		
	All subjects (*n* = 37)	= < 2 weeks (*n* = 8)	> 2 weeks (*n* = 29)
OD			
Self			
TDI	0.93**	0.91**	0.95**
I	0.88**	0.94**	0.85**
D	0.72**	0.83*	0.67**
T	0.61**	0.45	0.66**
Assisted			
TDI	0.87**	0.95**	0.83**
I	0.88**	0.92**	0.86**
D	0.68**	0.84*	0.64**
T	0.55**	0.96**	0.43

	ICC		
	All subjects (*n* = 84)	= < 2 weeks (*n* = 46)	> 2 weeks (*n* = 38)
HC			
Self			
TDI	0.61**	0.73**	0.40
I	0.51**	0.63**	0.29
D	0.40*	0.48*	0.35
T	0.30*	0.23	0.38
Assisted			
TDI	0.70**	0.68**	0.73**
I	0.69**	0.78**	0.49*
D	0.31*	0.02	0.55**
T	0.61**	0.43*	0.76**

The medium ± interquartile range of retest interval 18 ± 95 days, 3.5 ± 5.0 days for subjects retested within 2 weeks, and 58.0 ± 297.0 days for subjects retested in more than 2 weeks. ICC ≥ 0.9 indicates excellent reliability, 0.75 ≤ ICC < 0.9 indicates good reliability, and 0.5 ≤ ICC < 0.75 indicates moderate reliability

*p < 0.05, **p < 0.01

Bland–Altman plots with mean difference in self-assessment scores between first and second test session as well as the 95% LOA were shown to complement test interpretation [20]. For the TDI score, the mean difference was 0.88, 95% LOA ranged from − 7.40 to 5.65. For the I test the mean difference was − 1, 95% LOA ranged from − 5.51 to 3.51. For the D test the mean difference was − 0.06, 95% LOA ranged from − 3.38 to 3.26. For the T test the mean difference was 0.17, 95% LOA ranged from − 3.26 to 3.61 (See Fig. 2). The Error rates of TDI, T, D and I tests were also reported. Test error lower than the corresponding MCID was considered to be within an acceptable range. Based on the prospectively defined MCID (5.5 for TDI, 3 for I, 2.5 for T, and 3 for D), error rates of the assisted test were found as follows: 26% of TDI test, 29% of I test, 32% of D test, and 41% of T test in the total sample; 30% of TDI test, 25% of I test, 26% of D test, and 39% of T test in the HC group; 19% of TDI test, 38% of I test, 46% of D test, and 46% of T test in the OD group. Error rates for participants had test–retest intervals ≤ 2 weeks or > 2 weeks are also shown in Table 5.

**Table 5:** Percentage of participants that inter-test difference (error) reaches clinical significance (MCID)

	Error rate (|MD_test-retest_|> MCID)		
	All subject (*n* = 121)	= < 2 weeks (*n* = 54)	> 2 weeks (*n* = 67)
Total sample			
TDI	32 (26%)	16 (30%)	16 (24%)
I	35 (29%)	13 (24%)	22 (33%)
D	39 (32%)	15 (28%)	24 (36%)
T	50 (41%)	23 (43%)	27 (40%)

	Error rate (|MD_test-retest_|> MCID)		
	All subjects (*n* = 37)	= < 2 weeks (*n* = 8)	> 2 weeks (*n* = 29)
OD			
TDI	7 (19%)	3 (38%)	4 (14%)
I	14 (38%)	3 (38%)	11 (38%)
D	17 (46%)	3 (38%)	14 (48%)
T	17 (46%)	3 (38%)	14 (48%)

	Error rate (|MD_test-retest_|> MCID)		
	All subjects (*n* = 84)	= < 2 weeks (*n* = 46)	> 2 weeks (*n* = 38)
HC			
TDI	25 (30%)	13 (28%)	12 (32%)
I	21 (25%)	10 (22%)	11 (29%)
D	22 (26%)	12 (26%)	10 (26%)
T	33 (39%)	20 (44%)	13 (34%)

Error rate = Percentage of participants who had a test–retest difference greater than the minimum clinically important difference (MCID) of the corresponding Sniffin’ Stick subtest. MCID for TDI was 5.5, I was 3, for D was 3 and for T was 2.5 [22]. Maximally clinical acceptable error rate was defined as 40% [22]. For calculating error rate, the self-test TDI scores that ranged from 1 to 32 were transformed to the range of the standard TDI score, from 1 to 48 (TDItransformed = Tself × 2 + Dself × 2 + Iself). The self-T, and self-D scores that ranged from 1 to 8 were transformed to the range of 1 to 16 (Ttransformed = Tself × 2, Dtransformed = Dself × 2)

### Discrimination between OD and HC

Three-way rm-ANOVA analyses with Bonferroni post-hoc tests were conducted for Sniffin’ Sticks self-assessment. Group (HC and OD) was set as between-subject factor, session (1 and 2) and test (T, D, I) set as within-subject factors. No significant interactive effect of Session × Test × Group (*F*_(2,238)_ = 1.20, *p* = 0.30) was found, but significant interactive effects of Session × Group (*F*_(1,119)_ = 4.38, *p* = 0.04), Group × Test (*F*_(2,238)_ = 42.23, *p *< 0.01), and Session × Test (*F*_(2,238)_ = 13.41, *p* < 0.01) were observed. For Session × Group: HC group had better performance than the OD group in both sessions 1 and 2. For Group × Test: HC group performed better than the OD group in I, D and T tests. For Session × Test: subjects had significantly higher I score in session 2 than in session 1, but there were no significant differences between session 1 and 2 in D and T test scores. See Tables 6 and 7.

**Table 6:** Comparisons of the “Sniffin’ Sticks” self-test score between OD and HC group

	OD (*n* = 37)		HC (*n* = 84)		F			
	M	SD	M	SD	Session × Test × Group	Session × Group	Group × Test	Session × Test
Session 1								
I	8.19	4.00	13.04	2.08	1.20	4.38*	42.23**	13.41**
D	4.54	2.09	6.26	1.34				
T	2.84	1.60	3.99	1.37				
Session 2								
I	9.84	4.37	13.75	1.85				
D	4.73	1.79	6.26	1.34				
T	2.82	1.31	3.74	1.50				

OD = olfactory disorder, HC = healthy control. I = Identification, D = Discrimination, T = Threshold. M = mean, SD = standard deviation

*p < 0.05, **p < 0.01

**Table 7:** Simple effect analyses of Session × Group, Test × Group, and Test × Session

Session × Group	Session	Group		MD	SE	*p*
	1	HC	OD	2.57	0.30	< 0.01
	2	HC	OD	2.12	0.29	< 0.01

Test × Group	Test	Group		MD	SE	*p*
	I	HC	OD	4.38	0.51	< 0.01
	D	HC	OD	1.63	0.26	< 0.01
	T	HC	OD	1.04	0.23	< 0.01

Test × Session	Test	Session		MD	SE	*p*
	I	1	2	− 1.18	0.22	< 0.01
	D	1	2	− 0.10	0.17	0.57
	T	1	2	0.13	0.17	0.46

Method for multiple comparisons adjustment: Bonferroni; MD = mean difference; SE = standard error. OD = olfactory disorder, HC = healthy control. I = Identification, D = Discrimination, T = Threshold. M = mean, SD = standard deviation

*p < 0.05, **p < 0.01

Two-way rm-ANOVA with Bonferroni post-hoc tests were also conducted for self-assessed TDI total score. Group (HC and OD) was set as between-subject factor, while session (1 and 2) was set as within-subject factor. An interactive effect of Group × Session was found (*F*_(1,119)_ = 4.45, *p* = 0.04). In session 1, HC group (23.30 ± 3.24) performed better than OD patients (15.17 ± 6.55, *p* < 0.01). In session 2, HC group (23.76 ± 3.24) also had significant higher TDI scores than OD patients (17.39 ± 6.36, *p* < 0.01), although the difference decreased compared to that in session 1.

### Convenience and pleasantness of self- and assisted-assessment

**Fig. 3:**
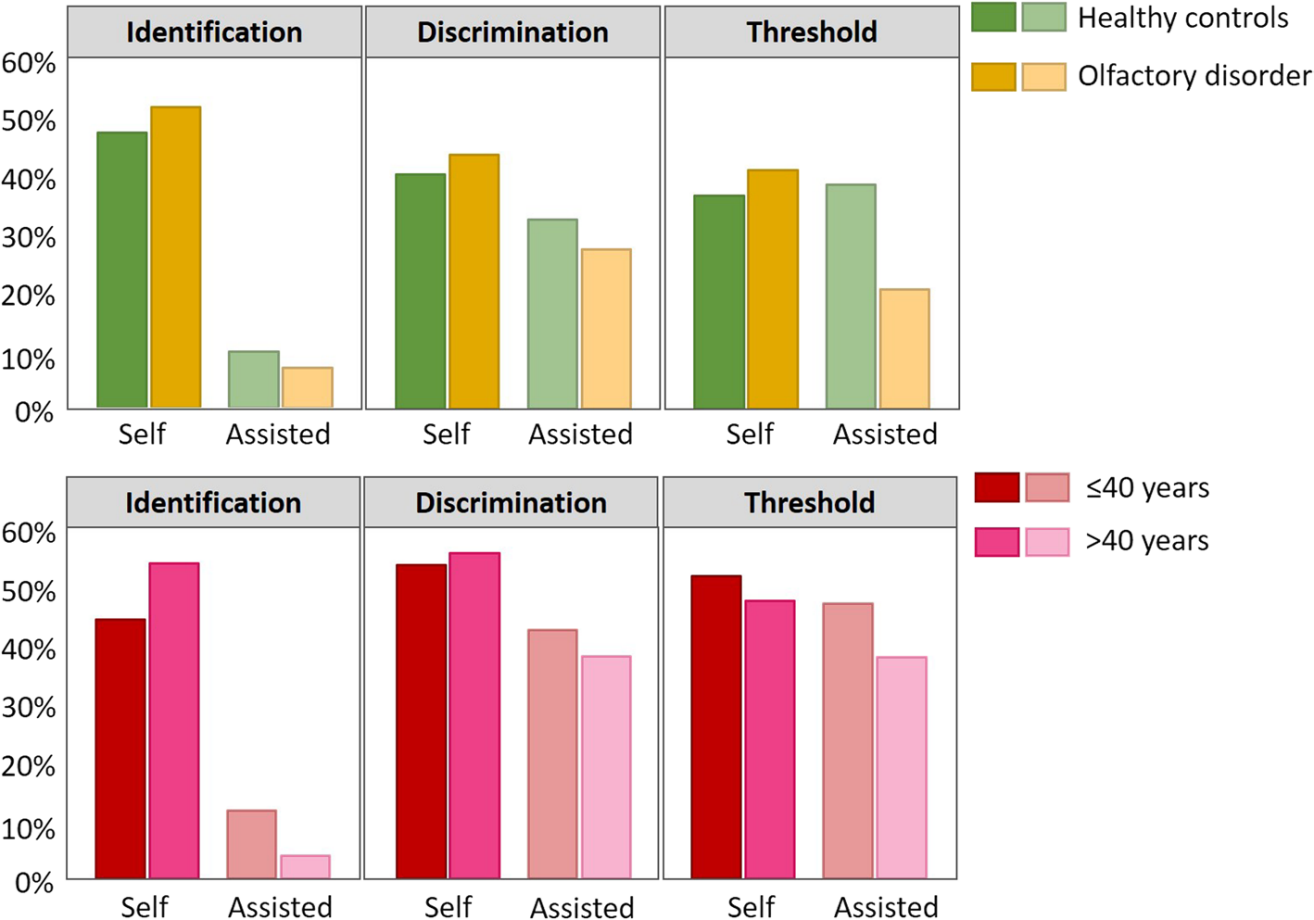
Convenience of self- and assisted-assessment in HC/OD group and in two age group. Percentage of participants favored self- or assisted assessment in tests for odor identification (I), odor discrimination (D) and odor threshold (T). Bar charts in green represent results from the healthy control group, yellow represents results from olfactory disorder patients, red represent results from healthy control group, and pink represents results from olfactory disorder patients

As shown in Fig. 3, more subjects generally considered self-assessment I, D tests as more convenient and pleasant in both HC and OD groups. As for T tests, in contrast to the OD subjects who favored test themselves, the HC subjects showed largely equivalent preferences to self- and assisted assessment.

Considering that age could be a possible factor influencing perception of convenience and pleasantness, we also reported it based on age. There was a higher percentage of subjects in both age groups preferring testing by themselves of all “Sniffin’ Sticks” subtests. See Fig. 3.

## Discussion

The present study establishes a self-administered procedure for comprehensive olfactory function assessment using the Sniffin’ Sticks for the purpose of medical personnel cost and time saving. Our results showed that Sniffin’ Sticks self-assessment was efficient in distinguishing between self-reported healthy subjects and OD patients. The identification subtest and the whole TDI test also exhibited good test–retest reliability and inter-test correlation to the well-validated Sniffin’ Sticks assisted assessment. However, the simplified Threshold and Discrimination self-subtests did not show adequate measurement properties when being applied by themselves.

For inter-test consistency, TDI and Identification self-tested scores were statistically consistent and exhibited moderate to excellent correlations with assisted-test in both HC and OD group, regardless of the simplified assisted test or the standard assisted test being used. Bland–Altman plots showed that the mean differences of TDI scores between self- and assisted tests were within a reasonable range that did not exceed the MCID value of 5.5 (ranged from − 0.01 to 2.10), which indicates that self-test were not different from assisted-test to a degree of having practical meaning. However, the 95% LOA of the Bland–Altman plot in OD group seemed to be wide and appeared to exceed the MCID, indicating that the inter-test difference of some cases was over the acceptable level. We, therefore, calculated the rates of the inter-test difference that exceeded the MCID value. Our results suggested that the between-test error rates of TDI and I scores were all far less than the maximally acceptable range of 40% in all groups (ranging from 1 to 19%). Taken these results together, the Identification and the whole Sniffin’ Sticks self-test exhibit a good consistency with the assisted test.

However, when it comes to the Threshold and Discrimination self-test, their correlations with assisted tests were much lower than the whole TDI test and I subtest, and even did not reach a minimally acceptable inter-correlation level of 0.4 in some subgroups. Furthermore, the inter-test error rates of T in HC group (41%) and the error rates of T (61%) and D (42%) tests in OD group who had standard assisted Sniffin’ Sticks testing were over the maximally acceptable range of 40%. This may imply that the simplified Sniffin’ Sticks D and T tests are only similar and closely relate to the assisted version when they are part of the overall test (TDI) rather than when they are separately used. A reason for this may be that the self- T and D tests were shorter than the standard versions, which may reduce the test precision.

We found a good to excellent test–retest reliability (ICC = 0.88–0.90) of the whole TDI test using the self-administered procedure no matter the test–retest intervals were short (≤ 2 weeks) or long (> 2 weeks) in the total sample. With regard to specific subtests, test–retest reliability of the I, D and T test could also reach acceptable to good levels (except on the T test for a test–retest time interval ≤ 2 weeks, ICC = 0.4). Overall, this indicates that the Sniffin’ Stick test is suited to be reliably self-administered.

However, when exploring OD and HC groups separately, the test–retest reliability showed a large discrepancy. For example, although test–retest reliability was excellent in OD subjects in the whole Sniffin’ Sticks test regardless of the test–retest time interval, and the reliability in self-administered testing was slightly better than the reliability in assisted-assessment. HC subjects only exhibited moderate reliability (ICC = 0.73) with short test–retest time interval, which decreased to non-significant when the test–retest time interval was over 2 weeks (ICC = 0.40). It is worth noting that the inter-individual olfactory function of included participants can affect the test–retest reliability [16]. That means in the present study, that the OD group including subjects with a wide range of olfactory function (hyposmia and anosmia) tended to exhibit higher test–retest reliability coefficients compared to the HC group including subjects with a narrower range of olfactory function (normosmia) [16, 26]. Such discrepancy could also be explained by the fact that participants in the OD group may have higher motivation to figure out their olfactory function and would be highly cooperative and attentive when completing tests, which may improve test–retest consistency [27]. Overall, these results indicated that self-administered test is more reliable in OD patients than in healthy participants.

In addition, although overall TDI score, I and D tests produced reliable results, T test reliability with test–retest time interval ≤ 2 weeks did not reach an acceptable level. Furthermore, for HC group, self- test was not as reliable as assisted assessment when test–retest interval was > 2 weeks. And although I and the whole tests had an acceptable reliability with test–retest interval ≤ 2 weeks, D and T tests did not exhibit a satisfying level of reliability. It seems that the simplified version of D and T tests currently are not reliable enough when applied in self-administered assessment. Again, this may be because the T and D tests were shorter than the standard version. As a rule, test reliability increases with duration of the test or the number of items tested, respectively [27–29]. Another reason may be that the number of patients included for the individual analyses was relatively low.

In addition to ICC values, we also described the Bland–Altman plot and calculated error rates of the Sniffin’ Stick self-test as supplements. 95%LOA of the Bland–Altman plot seemed to be wide. For example, 95%LOA of the I test ranged from − 5.51 to 3.51, appearing to exceed the I test MCID value 3. However, since the error rates of the TDI and I self-test were less than the maximally acceptable range of 40% no matter whether the long or short retest interval was investigated, and no matter whether HC or OD patients were studied (ranged from 19 to 38%), we could still argue that the whole TDI and I test–retest differences were within an acceptable range. However, the test–retest error rates of T and D test were much higher and even over the maximally acceptable range of 40%, indicating that the T and D self-test were not stable and reliable enough to be solely applied. As for the tendency of higher error rate of OD patients with a retest interval > 2 weeks than with a retest interval ≤ 2 weeks, this could be due to the improvement of olfactory function in OD patients with a long interval between two test sessions.

In our test cohort, “Sniffin’ Sticks” self-administered test could distinguish between OD and HC subjects, no matter whether the entire Sniffin’ Sticks battery was used or any of the subtests, and regardless of test sessions 1 or 2. This indicated that even if the test is self-administered by the subject, the test results can accurately distinguish patients from healthy individuals. In addition, we observed that subjects had higher scores in session 2 than session 1 of the I test. This implied an effect of practice in odor identification testing. Randomizing the sequences of odors tested and presenting items randomly could probably prevent such learning effects.

Measurement tools in a clinical context are perceived as more or less interesting, comfortable and tolerable. The patients’ experience is often a critical issue whether a tool could be widely used or not. In the present study, based on the participants’ subjective impression, more subjects preferred the self-administered method. This was similar in older and in younger participants, as well as in OD patients and healthy participants, with the exception of slightly higher preference for assisted T test in the HC group. It is worth noting that such preference differences were small and there was also a part of the subjects who reported no preference towards any of the two test methods. Hence, it could still be maintained that the self-administration of the Sniffin’ Sticks test was generally accepted and favored over the assisted test, or, as a minimum conclusion, that there was no major difference in terms of acceptance of the two forms of the test.

Overall, a series of analyses pointed to a similar tendency that the whole TDI and Identification self-tests appear to show good consistency and reliable measurement properties to the assisted-tests, while the simplified version of D and T tests currently are not reliable enough when separately applied in self-administered assessment, although they are acceptable to be self-administrated when they are parts of the whole TDI test. From the perspective of clinical practice, the Sniffin’ Sticks Identification test, which is easy-to-use and entertaining, is suitable to be solely applied for fast screening purposes by the participants themselves. The whole Sniffin’ Sticks self-test, with good measurement properties, is also suited to be used as an alternative in clinical practice, although some individual parts (D and T self-test) need to be improved before they can be used in general clinical practice.

Several limitations of the current study should be pointed out. First, as self-administered Sniffin’ Sticks Threshold does not work on their own, a computer program or an instructor is needed. And in our test cohort, there was an instructor to give some guidance to the subjects. The instructor guided the subjects to select the next test number of the pen once the subjects sniffed and selected an answer. Applying an adaptive computer program would be helpful in achieving a fully self-administered procedure. For example, the ‘‘Filemaker’’ based software ‘‘OLAF’’ guides the patient through any user-defined arrangement of the various portions of the test battery [30]. Other automated test systems have also been proposed [14, 22, 31, 32]. Moreover, the test cohort used the shorter versions of Discrimination and Threshold tests to save testing time and prevent subjects getting tired. However, aspects of our results implied that the shorter versions may not be suitable enough to be solely applied. To balance test precision and the patients’ attention to the task, testing the full-length self- and assisted test on a consecutively separate day for each visit may be worth trying in future studies. In addition, although the exact time required for the test was not recorded, it took approximately 45–60 min for each session, including a self- and assisted test. The self-administered part took approximately 10 min longer than the assisted part, as the participants had the chance to sniff as often and long as they wanted, whereas for the assisted part they were only allowed to sniff once, except for the odor identification part. When it came to the assessment of test–retest reliability the number of subjects in the respective subgroups was relatively small. This resulted in a sample size issue which reflected on the interpretation of the results for these subgroups.

## Conclusion

With good measurement properties, the Sniffin’ Sticks Identification test can be easily applied by the subjects themselves, and is therefore an easy-to-use alternative for olfactory screening testing. The simplified version of D and T tests, with restrictions, may not be ideal to be solely applied, but could be performed in a self-administered manner as part of the Sniffin’ Sticks overall test. The whole “Sniffin’ Sticks” self-test exhibits good measurement properties and appears to be a reasonable backup in clinical practice.

## Data Availability

The dataset used and analyzed during the current study is available from the corresponding author on reasonable request.
